# Running Opposes the Effects of Social Isolation on Synaptic Plasticity and Transmission in a Rat Model of Depression

**DOI:** 10.1371/journal.pone.0165071

**Published:** 2016-10-20

**Authors:** Marta Gómez-Galán, Teresa Femenía, Elin Åberg, Lisette Graae, Ann Van Eeckhaut, Ilse Smolders, Stefan Brené, Maria Lindskog

**Affiliations:** 1 Department of Neuroscience, Karolinska Institutet, Stockholm, Sweden; 2 Department of Neurobiology, Care Sciences and Society, Karolinska Institutet, Stockholm, Sweden; 3 Department of Physiology and Pharmacology, Karolinska Institutet, Stockholm, Sweden; 4 Department of Pharmaceutical Chemistry and Drug Analysis, Center for Neurosciences, Vrije Universiteit, Brussel, Belgium; Bilkent University, TURKEY

## Abstract

Stress, such as social isolation, is a well-known risk factor for depression, most probably in combination with predisposing genetic factors. Physical exercise on the other hand, is depicted as a wonder-treatment that makes you healthier, happier and live longer. However, the published results on the effects of exercise are ambiguous, especially when it comes to neuropsychiatric disorders. Here we combine a paradigm of social isolation with a genetic rat model of depression, the Flinders Sensitive Line (FSL), already known to have glutamatergic synaptic alterations. Compared to group-housed FSL rats, we found that social isolation further affects synaptic plasticity and increases basal synaptic transmission in hippocampal CA1 pyramidal neurons. These functional synaptic alterations co-exist with changes in hippocampal protein expression levels: social isolation in FSL rats reduce expression of the glial glutamate transporter GLT-1, and increase expression of the GluA2 AMPA-receptor subunit. We further show that physical exercise in form of voluntary running prevents the stress-induced synaptic effects but do not restore the endogenous mechanisms of depression already present in the FSL rat.

## Introduction

Depression is a mood disorder characterized by both emotional and cognitive symptoms. Despite the intense research in the field, the neurobiology of depression remains elusive, however emerging evidences place the glutamatergic system as central to the neurobiology and treatment of the mood disorders [1]. Our understanding of the etiology of the disease is limited to a list of risk factors where genetic predisposition and environmental risk factors, such as stressful life events, are thought to interact. If the stress-induced mechanisms of depression differs from the endogenous (genetic) factors is not clear.

Social isolation is known to be a strong stressor for both rodents and humans. In humans, social isolation is associated with higher risk of mental health problems, such as depressive and anxiety disorders [2, 3] and increased risk of mortality [4]. In rodents, social isolation induces anxiety- and depressive-like behaviors, aggression and memory impairments [5–7], whereas social interactions has been shown to be protective against stress-induced changes [8]. Recent work shows how neurobiological factors affected by stress can be studied to understand resilience to stress [9], an important way to understand psychiatric disorders [10]

Here we studied the effect of social isolation on the hippocampal glutamatergic system of a selectively bred strain of rats: the Flinders Sensitive Line (FSL). The FSL rat has been extensively used as a model for depression since it exhibits several depressive-like responses in behavioral tests [11–14]. The strain was originally created to be supersensitive to acetylcholine esterase [15], and it is possible that the increases sensitivity to cholinergic agents is associated with the increased sensitivity to stress displayed by these animals [16–18], but it should be noted that FSL rats also have deficiencies in many other systems, including dopaminergic and glutamatergic transmission [14]. Our lab have associated the depressed behavior in FSL rats with alterations in the hippocampal glutamatergic system, including impairments in synaptic transmission and plasticity (LTP), down-regulation of the glial glutamate transporter GLAST, and decreased levels of the NMDA receptor co-agonist D-serine [11]. These identified hallmarks in the FSL rat make them an optimal model to investigate the potential converging endogenous and stress-induced mechanisms of depression. Endurance exercise has been show to protect the brain from the stress [19], and previous work has shown that running partly reduces the depressive like behavior in FSL rats [20, 21]. Thus, we used voluntary running as an intervention that reduces depressive symptoms [22, 23] and found that running differentially affects the mechanisms affected by stress but not the endogenous mechanisms.

## Methods

The *Flinders Sensitive Line* (FSL) is a selectively bred rat line derived from Sprague-Dawley rats [15, 24]. The animals used in this study were bred at the Karolinska Institute and the Ethical committee for animal research in Stockholm, Sweden approved all animal experiments. The animals had free access to food and water and housed in a controlled environment of 12-h light/dark cycle.

10–12 weeks old male Sprague Dawley (SD) and FSL rats were divided in three groups: group-housed (GH; 3–4 rats per cage), individually housed (SI) and individually housed with free access to a running wheel (34 cm in diameter) for five weeks (SI+R). Running behavior was recorded every tenth minute by a computer-based data system with customized software and animal run on average 1250 ± 146 m per day. Note that in the SI group the running wheel was present and accessible to explore during the whole period but blocked for running, in order to not have differences in environmental enrichment between the two groups. After 5 weeks, animals were anesthetized with isoflurane and decapitated. One of the hippocampi was immediately frozen down to -80°C and kept for posterior molecular analysis. The second hemisphere was cut into 400 μm hippocampal slices and LTP induction protocol was performed in CA1 area as previously described [25].

### Electrophysiology

Briefly, slices were incubated in an interface chamber containing artificial cerebrospinal fluid (aCSF) (in mM): 130 NaCl; 3.5 KCl; 1.25 NaH2PO4, 24 NaHCO3, 2 CaCl; 1 MgCl and 10 glucose, pH 7.4) for at least 2 h and then transferred to the recording chamber. Field excitatory post-synaptic potentials (fEPSP) at Schaffer collateral (SC)-CA1 synapses were elicited at 10-s intervals with a bipolar concentric electrode (FHC, ME, USA) and a extracellular recording pipette (filled with regular aCSF) placed in the stratum radiatum. Input-output (I/O) curves were obtained. The stimulus intensity was set to approximately 60% of the intensity that triggered population spikes and was determined empirically for each cell. For measuring long-term plasticity (LTP) in the CA1 region, stimuli were applied every 60 s for at least 20 min before LTP was induced using three trains of high frequency stimulation (HFS; 100 pulses at 100 Hz applied at 20-s intervals). Synaptic strength was monitored for 60 min and calculated using the initial rising slope phase of the fEPSP. The data was normalised with respect to the mean fEPSP slope that was recorded during the last 20 min of the baseline period. I/O curves were constructed using the Prism 5 program (GraphPad software, Inc., USA) following software instructions: first the X values (Intensity) of the I/O curve data were transformed to log form and the Y values (response: fEPSP slope) normalized. Data was fit to a sigmoid curve defined in Prism and the best-fits values of the curves from the different experimental groups were compared statistically using the F test, which compares the difference in sum-of-squares with the difference you would expect by chance. The result is expressed as the F ratio, from which a P value is calculated.

### Western blotting

Rats were anesthetized with isoflurane, sacrificed by decapitation and the brains were quickly removed to dissect the hippocampi. The tissue was immediately sonicated in MAPK-buffer (containing Triton-X, SDS, Tris-HCl, NaCl, EDTA and H2O) with two types of protease inhibitors (1:100 from Sigma #P8340 and 1:10 from Roche #04693124001) and one phosphatase inhibitor (1:7 from Roche #04 906 837 001). The samples were centrifuged at 13.200 rpm during 10 minutes at 4°C and supernatant was kept for protein analyses. BCA colorimetric method was used to determine the total amount of protein obtained. Equal amounts of protein (30 μg) were loaded onto a NuPAGE 4–12% Bis-Tris gel, Novex (Life Technologies, Glasgow, UK) and transferred to PVDF membranes (0.45μm) Immubolon-FL (Millipore, Temecula, CA, USA). Detection was based on a fluorescent secondary antibody that was visualized using the LICOR (Lincoln, NE, USA) Odyssey infrared fluorescence detection system. The data were quantified using ImageJ software (NIH, Bethesda, MD, USA) normalizing the values with **β-**actin. The primary antibodies used were at the following concentrations: GluA1 (1:100; Millipore, Temecula, CA, USA), GluA2 (1:1000; Millipore, Temecula, CA, USA), GluN1 (1:500; SYSY, Gottingen, Germany), GluN2A (1:1000; Tocris Bioscience, Bristol, UK), GluN2B (1:1000; Abcam, Cambridge, UK), GLAST (1:5000; human EAAT1) (Abcam, Cambridge, UK) and GLT-1 (1:5000; human EAAT2) (Abcam, Cambridge, UK) and **β-**actin (1:10000; Millipore, Temecula, CA, USA).

### Enantioselective liquid chromatography—fluorescence detection

Tissue samples were prepared by sonicating fresh frozen hippocampi in 500 ml of 0.1M NHClO4. Each standard/sample was neutralized with an equal amount of 0.1M NaOH, and 9 ml of the neutralized standard/sample was derivatized using 9 ml of the o-phthalaldehyde/N-isobutyryl-L-cysteine mixture. The enantioselective chromatography experiments were performed using a Shiseido capcell pak MG C18 column (Analis, Namur, Belgium). The compounds were eluted by gradient elution with a mobile phase that was delivered at a rate of 0.17ml/min. The gradient elution was performed using mobile phase A (0.025M phosphate solution, pH 9) and mobile phase B (methanol:water 60:40). For fluorescence detection, a RF-10Axl Shimadzu spectrofluorometric detector was modified by introducing a 2-ml semi-microcell (Shimadzu, Duisburg, Germany). The derivatives were measured with excitation and emission wavelengths of 340 and 450 nm, respectively. The integration computer programme, Clarity (DataApex, Antec, Zoeterwoude, the Netherlands), was used to integrate the chromatograms. A scientist blinded to the rat strain performed all analyses.

### Statistical analysis

Statistical analysis was performed using the Prism 5 program (GraphPad software, Inc., USA). Statistical significance was tested using the unpaired two-tailed Student’s *t*-test and one-way ANOVA where applicable.

## Results

**Fig 1:**
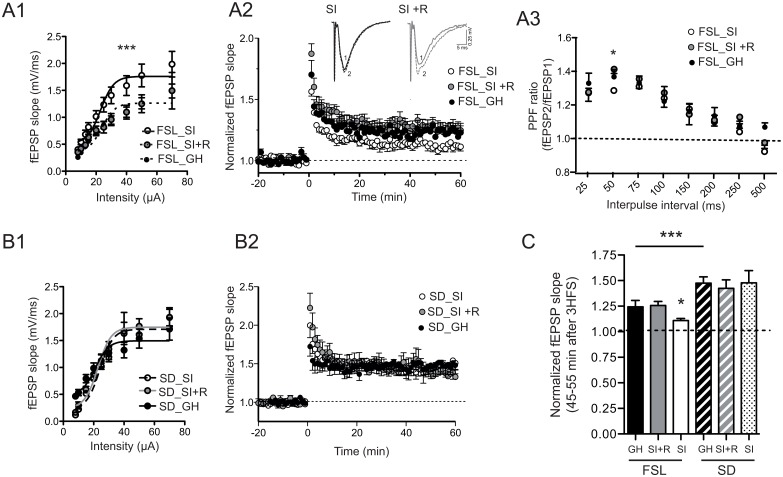
**A1-B1**. Input/output (I/O) curves obtained in CA1 area following Schaffer collateral stimulation in hippocampal slices from the three experimental groups of FSL rats (A1) and SD rats (B2). In all groups, the I/O curves were fitted to a sigmoid curve and the fitting resulting curve compared statistically according to the F test (see Material and Methods). According to this analysis, in FSL rats the sigmoid curve from the social isolation group (SI) differs significantly from the rest of the groups (GH and SI+R; ***P<0.0001), indicating an increase in synaptic transmission after social isolation. In SD rats (B1), each experimental group has its own sigmoidal function but they are not significantly different from each other. (N = 7 slices; 7 animals per group). **A2-B2**. LTP of Shaffer collateral (SC)-CA1 pyramidal cells synapses from FSL (A2) and SD (B2) hippocampal slices. Potentiation was measured as the slope of the fEPSP normalized to the average slope before high frequency stimulation (HFS). Time 0 indicates the onset of 3 HFS. (N = 7 slices, 7 animals per group). Top traces in A2 represent averaged fEPSPs from 5–0 minutes before stimulation (1) or 35–40 minutes after stimulation from representative slices taken from FSL:SI and FSL:SI+R groups. A3. Paired-pulse facilitation (PPF) obtained in CA1 area following collateral stimulation in hippocampal slices from the three experimental groups of FSL rats. PPF ratio: second fEPSP slope/first fEPSP slope. (N = 7 slices; 7 animals per group). **C**. Summary of the experiments shown in **A2-B2**. At 45–55 min after post-induction, potentiation in the FSL_SI group is decreased compared to FSL_GH group (*P<0.05). Running counteracts this effect (* P<0.05). SD rats show a higher level of potentiation compared to FSL rats (*** P<0.001 FSL_GH vs. SD_GH) no affected by social isolation or running. Bar-graph data in C represent means ± s.e.m. GH:group-housed, SI: social-isolated, SI +R: social-isolated with access to a running wheel.

Basal synaptic transmission from hippocampal slices from FSL and SD rats that had been groups housed (GH), socially isolated (SI) or socially isolated with access to a running wheel (SI+R) was measured using extracellular field recordings to generated input-output (I/O) curves to assess the differences in the responses to stimuli of a given intensity (from 10–70 μA) (Fig 1). In FSL rats, the I/O curves from the three groups were calculated independently and followed a sigmoidal function (r2 = 0.53, no difference between the groups), but while the GH and SI+R groups share the same curve (P = 0.74), the one from the SI group was significantly left shifted (Fig 1A1; ***P< 0.0001), indicating an increase in basal synaptic transmission. No significant changes were observed in any of the groups of SD rats (Fig 1B1).

We then showed that social isolation reduces long-term potentiation (LTP) in the FSL rats and, remarkably, running can prevent this reduction (Fig 1A2). At 45–55 min post-induction, potentiation in the GH and SI+R group was 121± 13% and 125 ± 6% respectively, versus 111 ± 8% in the SI group (*P < 0.05 vs. GH and SI+R; one-way ANOVA followed by Bonferroni’s multiple comparisons test; Fig 1C). As we previously reported (13), CA1-LTP was significantly decreased in FSL rats compared to SD rats (124.2± 8% vs. 148 ± 9%; ***P<0.001; one-way ANOVA followed by Bonferroni’s multiple comparisons test; Fig 1C). Importantly, we did not observe any effect of social isolation or running on plasticity (LTP) in the SD rats (Fig 1B1 and 1B2) confirming that the effect synaptic transmission and plasticity that we observe in the FSL rats upon social isolation is associated with their increased vulnerability to stress.

To understand if the changes in I/O-curve and LTP were associated with presynaptic changes we studied PPF in slices from FSL rats, applying two consecutive pulses with the same intensity but at different intervals (from 50–750 ms). We found a reduction in the PPF ratio only at the 50 ms interpulse interval in the SI group (*P<0.05; one-way ANOVA followed by Bonferroni’s multiple comparisons test; Fig 1A3), which was again reversed in the SI+R group.

**Fig 2:**
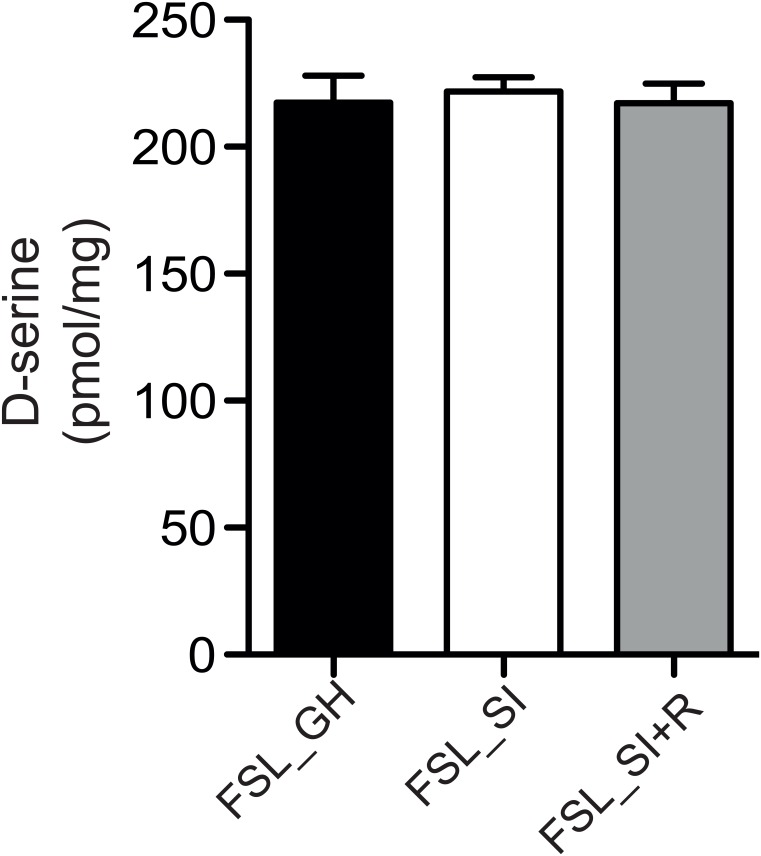
Quantification of the D-serine content in the hippocampal homogenates by enantioselective chromatography (N = 7–8 animals per group). Bar-graph represent means ± s.e.m. GH:group-housed, SI: social-isolated, SI +R: social-isolated with access to a running wheel.

Previously, we reported that the reduced LTP in FSL rats compared to SD rats was partially due to decreased levels of D-serine [25], a NMDA co-agonist known to enhance synaptic plasticity [26]. We therefore hypothesized that D-serine might be involved in the effect of social isolation on LTP. However, using enantioselective chromatography, we found similar D-serine levels in hippocampal homogenates from the three groups (Fig 2).

**Fig 3:**
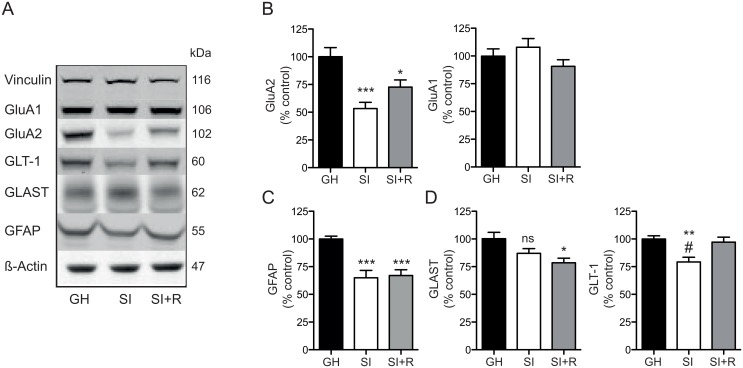
**A-C**. Representative western blots with their corresponding molecular weights (kDa) (**A**) and densitometry analysis for the different tested proteins: AMPA-R subunits: GluA1-2 (**B**), the astrocytic marker GFAP (C) and glial glutamate transporters: GLAST and GLT-1 (**D**) presented as percentage (%) of GH (control group) after normalization to **β-**actin or vinculin (loading control). Bar-graph data represent means ± s.e.m. (N = 6–8 animals per group). GH: group-housed, SI: social-isolated, SI +R: social-isolated with access to a running wheel. ***, * vs. GH; # vs. SI+R; one-way ANOVA followed by Bonferroni’s multiple comparisons test. GH:group-housed, SI: social-isolated, SI +R: social-isolated with access to a running wheel.

To further explore the mechanisms responsible for the changes in synaptic transmission and plasticity due to social isolation, and the effect of voluntary running, we analyzed the expression levels of several proteins involved in glutamatergic transmission (Fig 3). We did not find any alterations in any of the NMDA-R subunits (GluN1, GluN2A, GluN2B; data not shown). However, we found that social isolation induces a significant decrease in the protein expression level of the AMPA receptor subunit GluA2 (53.3 ± 5.6% of GH; ***P<0.0005 vs. GH; one-way ANOVA followed by Bonferroni’s multiple comparisons test; Fig 3B left), with no effect on the GluA1 subunit (Fig 3B right). Interestingly, running partially rescued the decrease in GluA2 protein levels (72.7 ± 6.5% of GH; *P<0.05 vs. GH; one-way ANOVA followed by Bonferroni’s multiple comparisons test) (Fig 3B left).

Next, we observed that social isolation decreased the levels of the glial fibrillary acidic protein, (GFAP) and that this protein remains decreased in the running group (65± 6.6% and 67.1± 5.2% of GH, respectively; ***P<0.0005; one-way ANOVA followed by Bonferroni’s multiple comparisons test) (Fig 3C). This is in agreement with findings from stress-induced depression [27] and opposite to the increase in GFAP levels observed in the FSL rats compared to the SD rats [25]. Giving these two opposing results we consider that more work is needed to interpret and understand the functional consequences of changes in GFAP levels. In addition, we also observed that the levels of the glial Glutamate Aspartate Transporter (GLAST), already reduced in FSL rats, showed a non-significant decreasing trend in the SI rats (87.1 ± 4.3% of GH; P = 0.08; one-way ANOVA followed by Bonferroni’s multiple comparisons test) that was further decreased by running (78.5 ± 4.2% of GH; *P<0.05 vs. GH; one-way ANOVA followed by Bonferroni’s multiple comparisons test) (Fig 3D left). In contrast, the protein expression levels of the other glial glutamate transporter (i.e., GLT-1) was significantly decreased in the SI group (79.2 ± 4.2% of GH; **P<0.005; one-way ANOVA followed by Bonferroni’s multiple comparisons test) and fully restored by running (#P<0.05 vs. SI; one-way ANOVA followed by Bonferroni’s multiple comparisons test) (Fig 3D right). GLT-1 is not affected in the FSL model compared to SD rats [25] but a decreased level of this transporter has been reported in the chronic mild stress model of depression [27].

## Discussion

The selectively bred FSL rat strain is extensively used as an animal model for depression, and one of its characteristic behavior is increased sensitivity to stress [13, 18]. One neurobiological mechanism associated with its depressed behavior is increased glutamate transmission and reduced plasticity in the hippocampus, conveyed by reduced levels of the glutamate transporter GLAST and reduced levels of D-serine [11]. Here we show that social isolation further enhances glutamate transmission and decreases synaptic plasticity in the FSL rats by targeting a different set of proteins involved in the glutamatergic system: GLT-1 and GluA2. Interestingly, we demonstrate that five weeks of voluntary running protects the brain against social isolation but does not affect the mechanisms already affected in the FSL rat.

A reduction in synaptic plasticity in the hippocampus is one of the most robustly proven neurobiological consequences of stress, whereas the change in basal glutamate transmission has been shown to increase, decrease or not change at all, depending on animal strain, stress protocol, age of the animals etc. [6, 28–30]. In our experiments, social isolation induced a potentiation of glutamate transmission (as assessed by a shift in the I/O curve). This increase is compatible with the observed reduction of the glutamate transporter GLT-1, since reduced levels of glutamate uptake will directly lead to increased levels of extracellular glutamate and a concomitant increase in glutamate transmission. A reduction of GluA2 seems more paradoxical, however, AMPA receptors lacking the GluA2 subunit have significantly different properties compared to GluA2 containing AMPA receptors, one of the most important being that they are permeable to calcium [31]. Indeed, it has been shown that activation of GluA2-lacking receptors induces a retrograde signal that enhances release probability at the presynaptic terminal [32]. Thus, a reduction of GluA2 could lead to increased glutamate transmission through an increase in release probability.

We have previously reported that the decreased hippocampal synaptic plasticity in the FSL compared to SD rats is due to decreased D-serine levels, a NMDA co-agonist known to enhance synaptic plasticity [11]. However, the effect of social isolation on synaptic plasticity is independent of D-serine, since we did not observed any differences in D-serine levels between these groups (Fig 2). Interestingly, we observed that although running restores LTP to the level of group-housed FSL, it does not increase LTP to the level of SD rats, suggesting that running does not affect LTP in the CA1 *per se*; rather it is protective from the deleterious effect of social isolation on LTP, maybe through the restoration of the synaptic proteins, GLT-1 and GluA2. On the other hand, social isolation has been shown to reduce hippocampal neurogenesis [33, 34] and one study in mice showed that running increases LTP in the DG by increasing neurogenesis in this area [35]. In this sense, we cannot exclude the possibility that the beneficial effect of voluntary running on SC-CA1 synapses may be mediated by increased hippocampal neurogenesis. In fact, since the hippocampus is a feed-forward network it is possible that the increase in number of young dentate granule cells after voluntary running can be accompanied by functional changes in the CA1 area. In fact, the antidepressant effect of both selective serotonin reuptake drugs and voluntary running has been suggested to occur through neurogenesis in the DG [20, 36–38].

The astrocytic proteins GLAST, GLT-1 and GFAP are differentially regulated by social isolation and running. This fact illustrates the complex effect of physical activity and stress on the brain (for review, see [39]). Both treatments have a direct effect on the HPA axis- and sustained physical exercise can reduce the response to stress on the HPA axis on several parameters, including reducing the levels of peripheral glucocorticoids [40, 41]. Paradoxically though, physical activity also activates the HPA axis functioning as a stressor [42, 43]. Finally, running also triggers other processes, such as oxidative stress, metabolic rate and blood flow, which affect the brain independently of the HPA axis illustrating the multiple and complex mechanisms underlying both the benefits and caveats of running [44–46]. The fact that social isolation and running have differential effect on specific astrocytic proteins in our model highlights this complexity and places the astrocytes as main actors in the mechanisms underlying the stress and running response in the brain.

In summary, our results show that in the FSL model of depression running counteracts stress-induced mechanisms of depression, but not endogenous mechanisms. Since most experimental depression models are stress-induced, these findings explain why physical exercise has consistently given good results in animal experiments [47, 48], while effects are less clear in patients [49]. They are also in line with the recommendation [50] that endurance activity is a good therapy for stress-induced depression, and that running can increase resilience to depression in individuals with a genetic predisposition for depression by acting on compensating mechanisms.

## References

[1] Sanacora G, Treccani G, Popoli M. Towards a glutamate hypothesis of depression: an emerging frontier of neuropsychopharmacology for mood disorders. Neuropharmacology. 2012;62(1):63–77. 10.1016/j.neuropharm.2011.07.036 21827775 PMC3205453

[2] Teo AR, Choi H, Valenstein M. Social relationships and depression: ten-year follow-up from a nationally representative study. PLoS One. 2013;8(4):e62396 10.1371/journal.pone.0062396 23646128 PMC3640036

[3] Santini ZI, Fiori KL, Feeney J, Tyrovolas S, Haro JM, Koyanagi A. Social relationships, loneliness, and mental health among older men and women in Ireland: A prospective community-based study. Journal of affective disorders. 2016;204:59–69. 10.1016/j.jad.2016.06.032 27337705

[4] Steptoe A, Shankar A, Demakakos P, Wardle J. Social isolation, loneliness, and all-cause mortality in older men and women. Proc Natl Acad Sci U S A. 2013;110(15):5797–801. 10.1073/pnas.1219686110 23530191 PMC3625264

[5] Chang CH, Hsiao YH, Chen YW, Yu YJ, Gean PW. Social isolation-induced increase in NMDA receptors in the hippocampus exacerbates emotional dysregulation in mice. Hippocampus. 2015;25(4):474–85. 10.1002/hipo.22384 25348768

[6] Djordjevic J, Djordjevic A, Adzic M, Radojcic MB. Effects of Chronic Social Isolation on Wistar Rat Behavior and Brain Plasticity Markers. Neuropsychobiology. 2012;66(2):112–9. 10.1159/000338605 22814229

[7] Zlatkovic J, Todorovic N, Boskovic M, Pajovic SB, Demajo M, Filipovic D. Different susceptibility of prefrontal cortex and hippocampus to oxidative stress following chronic social isolation stress. Molecular and cellular biochemistry. 2014;393(1–2):43–57. 10.1007/s11010-014-2045-z 24671494

[8] Liu X, Wu R, Tai F, Ma L, Wei B, Yang X, et al Effects of group housing on stress induced emotional and neuroendocrine alterations. Brain research. 2013;1502:71–80. 10.1016/j.brainres.2013.01.044 23380532

[9] Zanier-Gomes PH, de Abreu Silva TE, Zanetti GC, Benati ER, Pinheiro NM, Murta BM, et al Depressive behavior induced by social isolation of predisposed female rats. Physiol Behav. 2015;151:292–7. 10.1016/j.physbeh.2015.07.026 26209499

[10] King A. Neurobiology: Rise of resilience. Nature. 2016;531(7592):S18–S9. 10.1038/531S18a 26934522

[11] Gomez-Galan M, De Bundel D, Van Eeckhaut A, Smolders I, Lindskog M. Dysfunctional astrocytic regulation of glutamate transmission in a rat model of depression. Mol Psychiatry. 2013;18(5):582–94. 10.1038/mp.2012.10 22371047

[12] Femenia T, Magara S, DuPont CM, Lindskog M. Hippocampal-Dependent Antidepressant Action of the H3 Receptor Antagonist Clobenpropit in a Rat Model of Depression. Int J Neuropsychopharmacol. 2015. 10.1093/ijnp/pyv032 PMC4576519 25762718

[13] Magara S, Holst S, Lundberg S, Roman E, Lindskog M. Altered explorative strategies and reactive coping style in the FSL rat model of depression. Front Behav Neurosci. 2015;9:89 10.3389/fnbeh.2015.00089 25954168 PMC4404828

[14] Overstreet DH, Wegener G. The Flinders Sensitive Line Rat Model of Depression—25 Years and Still Producing. Pharmacological Reviews. 2013;65(1):143–55. 10.1124/pr.111.005397 23319547

[15] Overstreet DH, Russell RW. Selective breeding for diisopropyl fluorophosphate-sensitivity: behavioural effects of cholinergic agonists and antagonists. Psychopharmacology (Berl). 1982;78(2):150–5. 6817373 10.1007/BF00432254

[16] Markou A, Matthews K, Overstreet DH, Koob GF, Geyer MA. Flinders resistant hypocholinergic rats exhibit startle sensitization and reduced startle thresholds. Biol Psychiatry. 1994;36(10):680–8. 7880937 10.1016/0006-3223(94)91177-0

[17] Pucilowski O, Eichelman B, Overstreet DH, Rezvani AH, Janowsky DS. Enhanced affective aggression in genetically bred hypercholinergic rats. Neuropsychobiology. 1990;24(1):37–41. 2132639 10.1159/000119040

[18] Pucilowski O, Overstreet DH, Rezvani AH, Janowsky DS. Chronic mild stress-induced anhedonia: greater effect in a genetic rat model of depression. Physiol Behav. 1993;54(6):1215–20. 8295967 10.1016/0031-9384(93)90351-f

[19] Agudelo LZ, Femenia T, Orhan F, Porsmyr-Palmertz M, Goiny M, Martinez-Redondo V, et al Skeletal muscle PGC-1alpha1 modulates kynurenine metabolism and mediates resilience to stress-induced depression. Cell. 2014;159(1):33–45. 10.1016/j.cell.2014.07.051 25259918

[20] Bjørnebekk A, Mathé AA, Brené S. The antidepressant effects of running and escitalopram are associated with levels of hippocampal NPY and Y1 receptor but not cell proliferation in a rat model of depression. Hippocampus. 2010;20(7):820–8. 10.1002/hipo.20683 19623606

[21] Brené S, Bjørnebekk A, Åberg E, Mathé AA, Olson L, Werme M. Running is rewarding and antidepressive. Physiology & Behavior. 2007;92(1–2):136–40. 17561174 10.1016/j.physbeh.2007.05.015 PMC2040025

[22] Bjørnebekk A, Mathé AA, Gruber SHM, Brené S. Housing conditions modulate escitalopram effects on antidepressive-like behaviour and brain neurochemistry. The International Journal of Neuropsychopharmacology. 2008;11(08):1135–47. 18570703 10.1017/S1461145708008912

[23] Lapmanee S, Charoenphandhu J, Charoenphandhu N. Beneficial effects of fluoxetine, reboxetine, venlafaxine, and voluntary running exercise in stressed male rats with anxiety- and depression-like behaviors. Behavioural Brain Research. 2013(0). 10.1016/j.bbr.2013.05.018 23707245

[24] Overstreet DH. Behavioral Characteristics of Rat Lines Selected for Differential Hypothermic Responses to Cholinergic or Serotonergic Agonists. Behavior Genetics. 2002;32(5):335–48. 12405515 10.1023/a:1020262205227

[25] Gómez-Galán M, De Bundel D, Van Eeckhaut A, Smolders I, Lindskog M. Dysfunctional Astrocytic Regulation of Glutamate Transmission in a Rat Model of Depression. Molecular Psychiatry. 2012;AOP 2 28. 10.1038/mp.2012.10 22371047

[26] Henneberger C, Papouin T, Oliet SHR, Rusakov DA. Long-term potentiation depends on release of d-serine from astrocytes. Nature. 2010;463(7278):232–6. 10.1038/nature08673 20075918 PMC2807667

[27] Banasr M, Chowdhury GM, Terwilliger R, Newton SS, Duman RS, Behar KL, et al Glial pathology in an animal model of depression: reversal of stress-induced cellular, metabolic and behavioral deficits by the glutamate-modulating drug riluzole. Mol Psychiatry. 2010;15(5):501–11. 10.1038/mp.2008.106 18825147 PMC3347761

[28] Sanna E, Talani G, Obili N, Mascia MP, Mostallino MC, Secci PP, et al Voluntary Ethanol Consumption Induced by Social Isolation Reverses the Increase of α(4)/δ GABA(A) Receptor Gene Expression and Function in the Hippocampus of C57BL/6J Mice. Frontiers in Neuroscience. 2011;5:15 10.3389/fnins.2011.00015 21347217 PMC3039156

[29] Shin SY, Han SH, Woo RS, Jang SH, Min SS. Adolescent mice show anxiety- and aggressive-like behavior and the reduction of long-term potentiation in mossy fiber-CA3 synapses after neonatal maternal separation. Neuroscience. 2016;316:221–31. 10.1016/j.neuroscience.2015.12.041 26733385

[30] Kamal A, Ramakers GM, Altinbilek B, Kas MJ. Social isolation stress reduces hippocampal long-term potentiation: effect of animal strain and involvement of glucocorticoid receptors. Neuroscience. 2014;256:262–70. 10.1016/j.neuroscience.2013.10.016 24161282

[31] Hollmann M, Hartley M, Heinemann S. Ca2+ permeability of KA-AMPA—gated glutamate receptor channels depends on subunit composition. Science (New York, NY). 1991;252(5007):851–3. 10.1126/science.1709304 1709304

[32] Lindskog M, Li L, Groth RD, Poburko D, Thiagarajan TC, Han X, et al Postsynaptic GluA1 enables acute retrograde enhancement of presynaptic function to coordinate adaptation to synaptic inactivity. Proc Natl Acad Sci U S A. 2010;107(50):21806–11. 10.1073/pnas.1016399107 21098665 PMC3003060

[33] Stranahan A, Khalil D, Gould E. Social isolation delays the positive effects of running on adult neurogenesis. Nat Neurosci 2006;9(4):526–33. 10.1038/nn1668 16531997 PMC3029943

[34] Czéh B, Welt T, Fischer AK, Erhardt A, Schmitt W, Müller MB, et al Chronic psychosocial stress and concomitant repetitive transcranial magnetic stimulation: effects on stress hormone levels and adult hippocampal neurogenesis. Biological Psychiatry. 2002;52(11):1057–65. 12460689 10.1016/s0006-3223(02)01457-9

[35] van Praag H, Christie BR, Sejnowski TJ, Gage FH. Running enhances neurogenesis, learning, and long-term potentiation in mice. PNAS. 1999;96(23):13427–31. 10557337 10.1073/pnas.96.23.13427 PMC23964

[36] Yau S, Lau B, Tong J, Wong R, Ching Y, Qiu G, et al Hippocampal Neurogenesis and Dendritic Plasticity Support Running-Improved Spatial Learning and Depression-Like Behaviour in Stressed Rats. PLoS ONE. 2011;6(9). 10.1371/journal.pone.0024263 PMC3174166 21935393

[37] Malberg JE, Eisch AJ, Nestler EJ, Duman RS. Chronic Antidepressant Treatment Increases Neurogenesis in Adult Rat Hippocampus. The Journal of Neuroscience. 2000;20(24):9104–10. 11124987 10.1523/JNEUROSCI.20-24-09104.2000 PMC6773038

[38] Sahay A, Hen R. Adult hippocampal neurogenesis in depression. Nature Neuroscience. 2007;10:1110–5. 10.1038/nn1969 17726477

[39] Novak CM, Burghardt PR, Levine JA. The use of a running wheel to measure activity in rodents: Relationship to energy balance, general activity, and reward. Neuroscience & Biobehavioral Reviews. 2012;36(3):1001–14. 22230703 10.1016/j.neubiorev.2011.12.012 PMC4455940

[40] Adlard PA, Cotman CW. Voluntary exercise protects against stress-induced decreases in brain-derived neurotrophic factor protein expression. Neuroscience. 2004;124(4):985–92. 10.1016/j.neuroscience.2003.12.039 15026138

[41] Zheng H, Liu Y, Li W, Yang B, Chen D, Wang X, et al Beneficial effects of exercise and its molecular mechanisms on depression in rats. Behavioural Brain Research. 2006;168(1):47–55. 10.1016/j.bbr.2005.10.007 16290283 PMC2662337

[42] Hackney AC. Stress and the neuroendocrine system: the role of exercise as a stressor and modifier of stress. Expert Rev Endocrinol Metab. 2006;1(6):783–92. 10.1586/17446651.1.6.783 20948580 PMC2953272

[43] Fuss J, Ben Abdallah NMB, Vogt MA, Touma C, Pacifici PG, Palme R, et al Voluntary exercise induces anxiety-like behavior in adult C57BL/6J mice correlating with hippocampal neurogenesis. Hippocampus. 2010;20(3):364–76. 10.1002/hipo.20634 19452518

[44] Yancey SL, Overton JM. Cardiovascular responses to voluntary and treadmill exercise in rats. Journal of Applied Physiology. 1993;75(3):1334–40. 8226548 10.1152/jappl.1993.75.3.1334

[45] Colcombe SJ, Kramer AF, Erickson KI, Scalf P, McAuley E, Cohen NJ, et al Cardiovascular fitness, cortical plasticity, and aging. Proceedings of the National Academy of Sciences of the United States of America. 2004;101(9):3316–21. 10.1073/pnas.0400266101 14978288 PMC373255

[46] Coyle EF. Physical activity as a metabolic stressor. The American Journal of Clinical Nutrition. 2000;72(2):512s–20s. 10919953 10.1093/ajcn/72.2.512S

[47] Greenwood B, Fleshner M. Exercise, Learned Helplessness, and the Stress-Resistant Brain. Neuromol Med. 2008;10(2):81–98. 10.1007/s12017-008-8029-y 18300002

[48] Duman CH, Schlesinger L, Russell DS, Duman RS. Voluntary exercise produces antidepressant and anxiolytic behavioral effects in mice. Brain Res. 2008;1199:148–58. 10.1016/j.brainres.2007.12.047 18267317 PMC2330082

[49] Rimer J, Dwan K, Lawlor DA, Greig CA, McMurdo M, Morley W, et al Exercise for depression. Cochrane Database of Systematic Reviews. 2012;7. 10.1002/14651858.CD004366.pub5 22786489

[50] Firth J, Rosenbaum S, Stubbs B, Gorczynski P, Yung AR, Vancampfort D. Motivating factors and barriers towards exercise in severe mental illness: a systematic review and meta-analysis. Psychological medicine. 2016:1–13. 10.1017/S0033291716001732 PMC5080671 27502153

